# Strengthening the Paediatricians Project 1: The need, content and process of a workshop to address the Priority Mental Health Disorders of adolescence in countries with low human resource for health

**DOI:** 10.1186/1447-056X-9-4

**Published:** 2010-02-18

**Authors:** Paul SS Russell, Muttathu KC Nair

**Affiliations:** 1 Child and Adolescent Psychiatry Unit, Department of Psychiatry, Christian Medical College, Vellore 632 002, India; 2 Child Development Centre, Thiruvananthapuram Medical College, Thiruvananthapuram 695 011, India

## Abstract

**Objective:**

World Health Organization has identified *Priority Mental Health Disorders *(PMHD) of adolescence. To effectively address these disorders at the primary care level paediatricians have to be trained in the low-income countries, which often have paucity of mental health resources. We studied: (1) the need of psychiatric training required among paediatricians; (2) if the content and process of the model workshop suits them to identify and treat these disorders.

**Methods:**

Forty-eight paediatricians completed evaluation questionnaire at the end of a 3-day workshop on adolescent psychiatry. They participated in a focused group discussion addressing the areas in psychiatry that needs to be strengthened in these workshops, the changes in the content and process of the workshop to bolster their learning. Qualitative and descriptive analyses were appropriately used.

**Results:**

Training in adolescent psychiatry was considered necessary among the paediatricians at zonal level frequently to develop their private practice, treat psychiatric disorders confidently, make correct referrals, and learn about counselling. Prioritizing training from under and postgraduate training, integrate psychiatry training with conference, conducting special workshops or Continuing Medical Education were suggested as ways of inculcating adolescent psychiatry proficiency. Mental status examination, psychopathology and management of the PMHD were considered by the respondents as important content that need to be addressed in the program but aspects of behavioural problems and developmental disabilities were also identified as areas of focus to gain knowledge and skill. Appropriate group size, flexibility in management decisions to fit the diverse clinical practice- settings was appreciated. Lack of skills in giving clinical reasoning in relation to PMHD, time management and feedback to individuals were identified as required components in the collaborative effort of this manner. Providing delegates with basic information on adolescent psychiatry prior to the workshop was suggested to make the workshop more valuable.

**Conclusions:**

There is a need to expand training for paediatricians from various backgrounds in adolescent psychiatry to strengthen their clinical skills to address the PMHD at the primary-care level. The evaluation suggests that the design and collaborative approach evident in this programme have merit as a model for training paediatricians in adolescent psychiatry in countries with low human resource for health.

## Introduction

Adolescents are rarely perceived to be ill while infact they have significant morbidity and mortality related to accidents, chronic illness, sexual and mental health problems [1]. There is a high prevalence of psychopathology among adolescents [2] and the escalation of mental health admissions through adolescence is 14-fold by the age 15 [3]. World Health Organisation has identified the *Priority Mental Health Disorders *(PMHD) in adolescence and to combat these disorders it needs global effort at the primary health care provider level [4]. Identifying and effectively treating these disorders at the primary health care setting can significantly reduce the burden posed by them in countries where there is paucity of health resources, especially mental health resources [5].

In India, children and adolescents form nearly 37% of the total population. Mental illness and mental retardation in this age group is projected to increase to 32,142,337 in 2015 from 26,538,146 in 2001 [6]. The mental health resources available in India however is exceedingly inadequate- with a total psychiatric bed strength of 0.25, number of psychiatrists at 0.2, number of psychiatric nurses at 0.05, number of psychologists at 0.03, number of psychiatric social workers at 0.03 (all per 100 000 population) [7] - to address the increasing burden of care for this population. The burden posed by these disorders in India among this age group is as high [8] as in any other developing country because of the double burden [9], requiring collaboration between mental and health professionals in primary-care settings. In India three health professionals namely graduate doctors (with MBBS qualification), paediatricians (with diploma, diplomate or doctoral qualifications) and family medicine doctors (with diplomate or doctoral qualification) are the primary column of support for children for a myriad of physical and mental health needs and thus serve the clinical role of general practitioners. In India there are only a few family medicine professionals and paediatricians are popular among the common man as they are considered specialist over the graduate physician and over psychiatrist because of no stigma in consulting the paediatrician. Thus any attempt in developing countries to identify and treat these disorders at the primary health care setting will be incomplete unless paediatricians are involved in the process. Although the need to have collaborative pedagogy between paediatricians and child psychiatrists is widely recognized in the literature, there is a significant paucity on the practical approach to such training models [10,11].

These challenge are currently being addressed by many developing countries including India by training the paediatricians in identifying and treating the PMHD among adolescents. In India, National task Force on Family Life and Life Skill Education (NFLLSE) of the Adolescent Paediatrics Chapter of the Indian Academy of Paediatrics encourages training in adolescent mental health. As part of the Postgraduate Diploma in Adolescent Paediatrics by Child Development Centre and University of Kerala a three-day workshop was conducted to address the training needs of the paediatricians in identifying and treating the PMHD among adolescents.

This article explores the need, content and process of a three-day workshop for paediatricians in improving their adolescent psychiatry knowledge, ability to diagnose and treat the PMHD in the adolescent population using focused group approach.

## Methods

### Setting and participants

The workshop was conducted at the Child Development Centre, Thiruvananthapuram, for three days from the 19^th ^to 21^st ^of June 2006. Thiruvananthapuram district has a literacy rate of 88%. The University of Kerala is located in the city of Thiruvananthapuram. The state has 8 medical colleges teaching ayurveda, homeopathic and modern medicine. The public health system has a four-tier model with medical colleges forming the tertiary-care centres, followed by the district headquarters hospitals and taluk hospitals forming the secondary-care components of health care and the primary care centres providing the primary care to the state.

The participants were mostly paediatricians from academic background or general paediatric practice from different regions of India. These paediatricians were recruited to the Postgraduate Diploma in Adolescent Paediatrics by advertising in the national paediatric journals and newspapers by the University of Kerala and Child Development Centre. All those enrolled for the postgraduate diploma were selected for the workshop.

### Description of workshop

The workshop named as *Strengthening the Paediatricians Project *(SAPP) was conducted under the auspices of the NFLLSE of the Indian Academy of Paediatrics. The aim of the workshop was to introduce the Adolescent Psychiatry for paediatricians and thus refine the content and process of the workshop to effectively conduct similar workshops in the different regions of India and later in SAARC (South Asian Association for Regional Cooperation) countries. The workshop was for 27 hours spread over three days. The participants were divided in to five groups of 8-10 each and five psychiatrists invited by the NFLLSE facilitated each group. Among the facilitators, one was a child and adolescent psychiatrist and four were general adult psychiatrist proficient in child and adolescent psychiatric disorders. The general adult psychiatrists were recruited from medical colleges in Thiruvananthapuram and the child psychiatrist was invited from the Christian Medical College, Vellore in the neighboring state of Tamil Nadu. The facilitators were financially sponsored by the Child Development Centre. The outline of the workshop is briefed in Table1.

### Workshop content and facilitation

The opening session of the *first day *introduced the Child and Adolescent Psychiatry for paediatricians together with the aims of the workshop and the paediatricians' pre-workshop knowledge child and adolescent psychiatry was collected through five multiple choice questions. The next two sessions in the morning focused on the systems in mind (e.g. perception, thought, mood), and the related psychopathology identifiable in a mental status examination (e.g. hallucinations, delusions, elation) respectively. The two sessions in the afternoon focused on identifying the psychopathology with case vignettes and interviewing 'cases' for the psychopathologies role-played.

The two morning sessions of the *second day *focused on translating the previous days mental status examination findings of the 'cases' to the *International Classification of Diseases: Mental and Behavioral Disorders *(Clinical Descriptions and Diagnostic Guidelines) - Tenth version (ICD-10) [12] based diagnosis of PMHD that paediatricians will encounter as well as effectively treat with non-pharmacological interventions (e.g. learning disability, dissociative disorders, somatoform disorders, behavioural problems like self-injurious behaviour). The two afternoon sessions addressed the non-pharmacological intervention needed for some of the common mental disorders (e.g. antecedent- behaviour- consequence analysis and reinforcement technique for behavioural problems) discussed in the previous session.

The pre-lunch sessions of the *third day *addressed the PMHD that paediatricians will encounter that can be effectively treated with medication (e.g. depression, mania, psychoses, attention deficit hyperactivity disorder, autism) and pharmacotherapy of these mental disorders (e.g. antidepressants for depression, antipsychotic for psychosis and autism). During the post-lunch session, the concluding session, the same multiple choice questions to measure the post-workshop acquisition of knowledge was administered followed by a focused group discussion to explore the participants' needs, experiences, to consider key issues and feedback on the content as well as process of the workshop. The participants recorded their opinions descriptively after discussion with themselves and these transcripts were used for qualitative analysis. The number of hours spent in each session is concised in Table 1.

**Table 1:** Strengthening the Paediatricians Project workshop outline

Morning	Afternoon
Day 1 Opening session: Pre-workshop assessment (1/2 hour) Session 1: Phenomenology (2 hours) Session 2: Psychopathology (3 hours)	Session 3: Case vignettes for identifying psychopathology (2 hours) Session 4: Role-play for identifying psychopathology (2 hours)
Day 2 Session 1: Introduction to ICD-10 (1 hours) Session 2: Making ICD-10 diagnosis using the psychopathology identified in the role-plays previously (4 hours)	Session 3: PMHD requiring pharmacotherapy (3 hours) Session 4: Psychotherapy for PMHD (3 hours)
Day 3 Session 1: PMHD requiring psychotherapy (3 hours) Session 2: Pharmacotherapy for PMHD (3 hours)	Closing session: Post-workshop assessment Focused group discussion on the need, content and process of the workshop (1/2 hour)

The *content facilitation *was done by psychiatrists (facilitators) who were proficient in asking, clarifying the meaning of various systems and phenomenology in mind, clinically identifying the psychopathology, in encouraging paediatricians to integrate basic and clinical sciences by sharing their experiences with paediatricians. The focus of the content of the workshop was to identify and treat the disorders, and therefore the tutors did not emphasize on the impairment raised by the 'cases' as impairment related management was deemed difficult to be addressed by paediatricians during their busy real life schedules.

### Workshop process and facilitation

The workshop always used an initial interactive-dydactic teaching of the content by an academic psychiatrist with 13 years of clinical as well as teaching experience in childhood and adolescent mental illnesses. The dydactic teaching was using audio-visual materials. After this dydactic process the participants interacted among themselves and with this psychiatrist to clarify their queries. Following this interactive-dydactic phase, the participants were divided in to 5-6 small groups and different case vignettes were provided to each group to work on. A participant from each group was encouraged to present the findings on the case vignette with the help of the respective group. Once adept in identifying the psychopathology and diagnosing the common mental disorders presented in the case vignettes, they progressed to participating in role plays. The facilitating psychiatrists participated in the role plays as 'cases' depicting the priority mental disorders (this was essential to avoid using real cases of adolescents with mental illnesses that is fraught with ethical concerns) among adolescents. The cases role played were dissociative disorders, somatoform disorders, depression, mania, psychoses, attention deficit hyperactivity disorder, autism and behavioural problems like self-injurious behaviour. The paediatricians were encouraged to conduct diagnostic interview of the 'cases'. These interviews were videographed and all psychopathology portrayed were recorded by paediatricians to make an ICD-10 diagnosis and management protocol.

### Evaluation

Immediately following the workshop, participants were requested to complete an evaluation questionnaire following discussions. The evaluation questionnaire, which combined open and closed questions, sought participants' feedback about the need, content and process of the workshop. Forty-eight evaluation forms were completed. Seven participants (14.6%) left the workshop immediately prior to the closing session. This was unexpected and apparently not triggered by anything more than a need to catch commuter and intercity trains. In addition, each of the five facilitators offered their reflections about its effectiveness and their involvement. Firstly, for a global understanding of the content in each paediatrician's descriptive writings, the answers were first thoroughly read in extenso. Paediatricians' statements were coded into general categories, was further restructured and refined if any new categories were found and thus main themes were condensed. These tabulations eventually made were to encompass an overall picture of paediatrician's statements. Data was further reviewed to identify common responses, search for singularities and patterns, develop description of concepts, resolve key themes and develop accounts to explain interpretations. Deviant cases of statements not fitting the main themes were identified. Following these steps the descriptive and interpretative summaries were extracted and presented.

## Results

### Participant characteristic

Of the 48 respondents completing the pre workshop form, 77.3% were female and 22.7% were male with an age range of 29 to 58 years. Thirty one of them were Doctor of Medicine in paediatrics (MD), 9 Diploma in Child Health (DCH), 8 were medical graduates (MBBS) with special interest in paediatrics and wanting to pursue paediatric specialistion. The majority of the participants were practicing paediatricians and some were academicians (from tutors to professors). Among the participants the experience in treating paediatric disorders ranged from 1 to 23 years and a mean of 4.04(3.9) years. None of them had undergone child and adolescent psychiatry training as part of undergraduate, post-graduate and continuing professional development. It was notable that all of the participants have come across psychiatric disorders in their clinical practice.

### Exploring the need, content and process of workshop

The five inter-related themes that are key to understanding the need, content and process of this workshop to strengthen the pedaitricians were: What are the reasons for attending the workshop? Who should be the participants? What should be the content of the workshop? What should be the process of the workshop? And how to improve the workshop? The subthemes and number of participants' response are summarised in table 2 (refer to additional file 1).

### What are the reasons for attending the workshop?

Participants' reasons for attending the workshop were diverse and included a strong interest to develop their private practice, treat the childhood and adolescent psychiatric disorders, make referrals with correct diagnosis, wanting to know about counselling and ways of training junior paediatric faculty and postgraduates. One participant noted that he needed to help those children who have mental illness like what he had in his childhood.

### Who should be the participants?

Several participants recommended that attendance at the workshop becomes a mandatory element in the training of all child health care professionals namely the practicing paediatricians, academic paediatricians and under graduate as well as postgraduate trainee with the need to initially prioritize training for those most likely and frequently to be involved in children with abnormal behaviours. However, one participant noted that this should include paediatricians and also psychiatrists as it decreases inter-professional tension and enhance collaboration.

### What should be the approach for integrating child and adolescent psychiatry learning?

Almost every participant attending the workshop indicated that the undergraduate medical training could have and postgraduate training in paediatrics should have a training module in adolescent psychiatry as it influences the overall approach these youngsters can have in their later work. The next option will be to integrate the adolescent psychiatry training program in to the national, zonal conference or as special workshop in courses like the adolescent paediatric program and the last option is to conduct Continuing Medical Education (CME) Programs on the topic but was felt CME are not effective as often they are perceived as teaching programs with mediocre quality.

### What should be the content of the workshop?

There was considerable agreement that mental status examination, psychopathology and management of the PMHD were important. However, the overwhelming need was to address individual minor mental health disorders like breath-holding spells and stammering that are more commonly diagnosed and requested by parents in a general paediatric practice. Developmental disabilities like mental retardation and autism also were areas that the paediatricians wanted to gain knowledge and skill.

### What should be the process of the workshop?

The participants appreciated the summarizing of the various sessions to enable them globally process the overview of the workshop and thus consolidating broad topics like psychopathology, ICD-10 diagnosis and management. Using of videos to learn the different clinical aspects of adolescent psychiatry was valued. The groups' sizes were perceived as small enough for several participants to express their views and large enough for generating differential diagnosis as well as management plans. The flexibility in management decisions to fit within diverse settings (academic to primary-care settings) was mentioned as empowering them to deliver the newly acquired skills. The general professional attitude and specific professional skills demonstrated by the tutors were appreciated but lack of skills in giving clinical reasoning in relation to PMHD and managing group time as well as giving feedback to individuals within the group was pointed as major pit-falls in the training process.

### What are the improvements you would suggest to future workshop?

Most participants recorded at least one response to how the workshop could be improved. The single most frequently cited issue related to the venue accepting the physical comforts they had but pointing out that they space was just about sufficient when the entire group was divided to solve their clinical cases. Also, the need to have centres at different zones to help paediatricians in different regions in India was highlighted. Employing facilitators with psychiatry skills and provide delegates with basic information about adolescent psychiatry prior to the workshop could make the training more valuable.

## Discussion

This project has several implications for child and adolescent psychiatric education for paediatricians.

Firstly, training in child and adolescent psychiatry was considered as a necessity among the paediatricians from different regions of India. They suggested that such programs should be held at the zonal level of Indian Academy of Paediatrics and as a prelude zonal centres in these regions should be identified to conduct these workshops, preferably by the *Academy*. The paediatricians recognized the need to incorporate training in child and adolescent psychiatry in a medical undergraduate's curriculum to 'catch them young' in their training and as a priority in postgraduate paediatric training in India. For those paediatricians who have already completed their postgraduate training, it was thought that adolescent psychiatry training should be given either in the form of Continuing the Medical Education (CME) modules or special workshops.

Secondly, the content and process of the workshop programme was seen to be effective in terms of facilitating active learning and enabling achievement of learning needs. The participants although acknowledged the need to understand the basic psychiatric skills like mental status examination and identifying the psychopathology. However, they wanted to focus more on the ICD-10 based diagnosis and management than the mental status examination or psychopathologies. Another explicit demand during the workshop discussions was the need to add behavioural problems like breath-holding spells and stuttering in the content of the workshop. When it was pointed out that these behavioural problems are not priority mental health disorders it was held by many paediatricians that it would improve their practice. Similar felt needs of the paediatricians, to include management of behavioural problems in such workshops as a topic, have been expressed and documented in other contexts as well [13].

Thirdly, in our workshop the simulated patient/relative scenarios, using role-plays and videos, were considered the most effective teaching process. Other programmes using such simulated patients/relatives have confirmed the value of this technique in promoting learning, which is perceived as powerful and real in teaching mental health [14,15]. We therefore suggest that those involved in the curriculum preparation and training of paediatricians should consider this type of approach to facilitate acquisition of the requisite child and adolescent psychiatric skills to enable effective diagnosis and management of the priority mental health disorders.

Finally, a particular strength of the programme was the collaborative approach, evidenced by the participation of facilitators and participants from the disciplines of psychiatry and paediatrics. This reflected meaningful collaboration and provided positive role modelling for participants in recognizing the contribution of different professionals necessary in achieving a successful outcome for PMHD at the primary-care level. This workshop demonstrated a shared learning approach and is perhaps an example of the approach being increasingly encouraged at government level in other countries [16,17]. Previously too, it has been documented that child and adolescent psychiatrist working in collaboration with paediatricians and training paediatricians in mental health interview and evaluation techniques, recognition and diagnosis of behavioural has been found to increase mental health diagnoses in paediatric practices [18]. *Strengthening the Paediatricians Project *has the necessary elements for a collaborative approach between paediatricians and psychiatrists like mentioned previously [18]. Therefore, our workshop model if adopted in low-income countries can act as one approach in preparing paediatricians to respond to the increasing number of adolescents with priority mental health disorders.

The main caveat of the study is that this project used a relatively simple 'end of programme method' of evaluation. While this has yielded some helpful data we recognize the benefits of a pre-post workshop design, to ascertain more fully the effectiveness of the training intervention. Pre-attendance skills analysis checklists could be sent to participants when they register for the training event, with a follow up self-analysis on completion of the programme. Some participants supported the inclusion of video-recording as tool to aid analysis of the entire workshop. Several studies have shown the value of video-recording and its use merits serious consideration to enhance overall programme design [19,20]. Also, the cost of conducting the workshop was not studied and this could have helped those who want to replicate this workshop. Finally, the participants recorded their opinions descriptively in writing after discussing among themselves and these transcripts were used for qualitative analysis. Thus this group process had only some of the components of a focused group discussion in the qualitative sense.

In conclusion, this paper has reported an evaluation of training workshop designed to enhance the adolescent psychiatry knowledge and clinical skills of paediatricians. The workshops were delivered using a pragmatic approach, and in collaboration by facilitators from psychiatry background. There is a need to expand training opportunities for child health professionals from various backgrounds in adolescent psychiatry to ensure that they develop the knowledge and acquire the clinical skills to enable addressing of the priority mental health disorders at the primary-care level. The evaluation suggests that the design and collaborative approach evident in this programme have merit as a model for training paediatricians in child and adolescent psychiatry.

To enhance learning further, we plan to improve the quality of the workshops by including diversity in training formats quantity of training by increasing the venues and faculty.

## Abbreviations

CME: Continuing the Medical Education; DCH: Diploma in Child Health; ICD-10: International Classification of Diseases: Mental and Behavioral Disorders (Clinical Descriptions and Diagnostic Guidelines) - Tenth version; MBBS: Bachelor of Medicine and Bachelor of Surgery; MD: Doctor of Medicine; NFLLSE: National task Force on Family Life and Life Skill Education; PMHD: Priority Mental Health Disorders; SAARC: South Asian Association for Regional Cooperation; SAPP: Strengthening the Paediatrician Project

## Competing interests

The authors declare that they have no competing interests.

## Authors' contributions

PSSR was involved in the conception, designing, data analysis and interpretation, drafting and approving the final version. NMKC was involved in the conception, drafting and revising the final draft. All authors read and approved the final manuscript.

## Supplementary Material

Additional file 1 **Table S1**. Summary of the themes, sub-themes and responses of the paediatricians in the focused group discussion. Click here for file
