# Supplementary material for: Strengthening the Paediatricians Project 1: The need, content and process of a workshop to address the Priority Mental Health Disorders of adolescence in countries with low human resource for health
Source: Asia Pac Fam Med. 2010 Feb 18;9(1):4. doi: 10.1186/1447-056X-9-4 (PMC2836313; doi:10.1186/1447-056X-9-4)
Supplement: Additional file 1 — Table S1. Summary of the themes, sub-themes and responses of the paediatricians in the focused group discussion. [file 1447-056X-9-4-S1.DOC]

Table 2. Summary of the themes, sub-themes and responses of the paediatricians in the focused group discussion.

| *Themes* | *Subthemes* |  | *Summary and number of response by paediatricians* ^a^ |
| --- | --- | --- | --- |
| What is the reason for attending the workshop? | 1. Development of practice 2. Training of trainees 3. Need to refer 4. Wanting to help |  | - - *“to enhance my ability to manage emergency situations in my practice”* (N=29)   - *“to learn to integrate psychological aspects of child and family health into the busy acute training programme”* (N=6)   - *“ was wanting to know about the skills of communication, family therapy and management of behavioural and emotional issues”* (N=11)   - “*I have been trying to find ways of formalizing mental health training for paediatricians”* (N=3)   - “t*o participate in the development of paediatric psychological training”* (N=1)   - *“to try and improve our in-house teaching of psychological factors in paediatrics”* (N=2)   - *Want to improve my referral network* (N=8)   - *I have a long standing interest and involvement in the treating children and adolescents with mental illnesses because of my own illness* (N= 17) |
| Who should be the focus of training? | 1. Practicing paediatrician 2. Academic paediatrician 3. Trainees |  | - - *All paediatricians, as such workshop decrease the, what shall I say, the fear they have about psychiatric illnesses and lack of confidence to diagnose and treat such problems* (N=19)   - *May be my other faculty should also attend…*(N= 2)   - *Postgraduates to learn about adolescent psychiatric issues and them in turn can inculcate the culture among undergraduates* (N=11) |
| What should be the approach for training? | CME  Part of national/ zonal conferences  Special workshop  UG/PG training module |  | - - *I think there should be CMEs for all paediatricians and interested ones can enroll* (N=9)   - *CME are good if it is conducted periodically and after knowing what majority of paediatricians want* (N=3)   - *Workshops in national or zonal conference will enable better participation but time may be a problem* (N=31)   - *Workshop as part of adolescent paediatric like course will ensure paediatricians wanting to know adolescent psychiatry better and that should be the approach* (N=7)*.*   - *Psychiatry is an important area but neglected and it should be taught by including at different levels of undergraduate and postgraduate teaching* (N=40)*.* |
| The content of workshop | Mental status &  Psychopathology  Psychiatric disorders  Management |  | - - *Learning about mental status examination was useful.* (N= 39)   - *Mental retardation should be included; I felt almost cheated when I spoke to you about the need to know more about it and you said no to the topic.* (N= 17)   - *Please include breath-holding spells.* (N=2)   - *ADHD is a major problem and it should be always be part of the workshop.* (N= 12)   - *I have seen children with pain, I think with hindsight that they may have had somatoform disorder; I wish you would continue with that topic.* (N=2)   - *Managing sleep disorders are some thing I need to know otherwise my possibility of sleeping in the nights is remote!* (N=1)   - *Self-injurious behaviour topic is very important for clinicians and I feel confident of treating that and this topic will help many pediatricians.* (N=7)   - *Autism management had many details and all I want to know is what is the one medicine I need to prescribe; now I understand the choice of medication is target symptom based.* (N=13)   - *Include the management of stammering.* (N=1)   - *Any disorder, just tell us the most important symptoms that form a diagnosis and one medication to treat that.* (N=17) |
| The process of workshop | Group size  Enhancement of global and linear learning  Flexibility to suit different settings  Skills of tutors  Deficits of tutors |  | - *… found the groups for sessions were small enough to for several participants to express their views and large enough for diverse diagnosis and management related problem.* (N=16) - *Summarizing the opening session and other sessions in helping me see the link between different sessions.* (N=26) - *Using didactic teaching, case vignettes, videos and role-plays for building the knowledge and skills was useful and I can be duplicated it in my department.* (N= 37) - *The tutors were regular in attendance, were punctual and showed enthusiasm for the tutorial process.* (N=11) - *They were proficient in keeping the group on track, in giving feedback to the group, and in helping the group to function.* (N=9) - *The tutors did not give students adequate direction in the clinical reasoning process.* (N=17) - *… facilitators were less proficient in managing group time and in giving feedback to individuals within the group.* (N=4) |
|  |  |  |  |
| Improvement to workshop | Venue  Improve facilitation  Prior information |  | - *Groups were too large for their room and for 4-5 groups, their presence in the same large room impeded discussion.* (N=7) - *Workshops have to be conducted across the country at least one in each zone of Indian Academy of Paediatrics…* (N=25) - *.. stronger facilitation in some groups to ensure all participants understood and participated in the group discussions.* (N=6) - *Provide delegates with basic information prior to the workshop (while sending other details about accommodation etc) could have improved the quality of the final focused group discussions.* (N=29) |

^a^= total numbers of responses does not add-up to the total number of participants as there were overlapping reasons.
